# Determination of the Pressure Dependence of Raman Mode for an Alumina–Glass Pair in Hertzian Contact

**DOI:** 10.3390/ma15238645

**Published:** 2022-12-04

**Authors:** Karl Delbé, Cyril De Sousa, François Grizet, Jean-Yves Paris, Malik Yahiaoui

**Affiliations:** Laboratoire Génie de Production (LGP), Université de Toulouse, INP-ENIT, 47 Avenue d’Azereix, 65000 Tarbes, France

**Keywords:** Hertzian contact, Raman shift, pressure, alumina, soda–lime glass

## Abstract

Optimising the performance of materials requires, among other things, the characterisation of residual stresses during the design stage. Raman spectroscopy offers access to these residual stresses at the micrometre scale when this inelastic light scattering is active in these materials. In this case, the relationship between the Raman mode shift and the pressure must be known. High-pressure cells with diamond anvils or bending instruments coupled to Raman spectrometers are habitually used to determine this relationship. In this article, we propose a new method that involves a Hertzian contact to obtain this relationship. A device that compresses an alumina ball against a transparent glass plane is connected to a Raman spectrometer. Under these conditions, the contact pressure can be as high as 1.5 GPa. The contact between the glass plane and the ball is observed through a diaphragm. Several hundred Raman spectra are recorded depending on the contact diameter. The spectral profiles obtained represent the shift in the Raman modes of alumina and glass along the contact diameter. Hertz’s theory accurately describes the pressure profile as a function of position for elastic materials. Therefore, the contact diameter can be measured by fitting the spectral profile with a function identical to the Hertz profile. We then deduce the maximum pressure. Next, the calculated pressure profile along the contact diameter is correlated with the spectral profile. We obtain a pressure dependence of the Raman mode with a coefficient equal to 2.07 cm${}^{-1}$/GPa for the Eg modes of alumina at 417 cm${}^{-1}$, which is in good agreement with the literature. In the case of glass, we refine the measurement of the Q3 mode shift at 1096 cm${}^{-1}$ in the studied pressure range compared to the literature. We find a coefficient of 4.31 cm${}^{-1}$/GPa. This work on static contacts opens up promising prospects for investigations into dynamic contacts in tribology.

## 1. Introduction

Improving the performance of a ceramic material requires a deeper understanding of these materials. This new knowledge makes it possible to optimise the manufacturing processes and prolong its products’ operating conditions [1,2,3]. Determining residual stresses in the volume and on the material’s surface is essential to achieve this goal. Indeed, residual stresses can significantly affect a ceramic part’s mechanical properties, reliability and service life—either beneficially or detrimentally [4]. In the case of parts subjected to contact pressure or tribological stress, it becomes essential to compare these residual stresses before and after the tests [5,6,7,8]. Ideally, it would also be possible to determine the pressure distribution and its evolution throughout the contact at the interface under static or dynamic conditions. However, this is a difficult task due to the confinement and opacity of the contact.

Several techniques are available to quantify residual stresses. We can mention, among others, X-ray diffraction (XRD), neutron diffraction, indentation and nuclear magnetic resonance, fluorescence spectroscopy [9,10,11,12], micro-displacement laser transducer [13] and photoluminescence piezo-spectroscopy (PLPS) [14,15,16].

The analysis of ceramics by Raman spectroscopy offers interesting characterisation perspectives for mechanics [13,17,18] and tribology [16,19,20]. The spectrometer laser can reach micrometres or even sub-micrometre probe sizes when focused on a sample using a microscope. In Raman spectroscopy, the inelastic scattering of monochromatic radiation allows the detection of vibrational groups. It also permits the identification of the microstructure. Moreover, Raman scattering is also sensitive to stress variations. Thus, the wave number increases with compression and vice versa [21]. Raman spectroscopy has proven its effectiveness in estimating residual stresses [9,16,22]; however, it is necessary to determine the relationship between the Raman shift and the pressure depending on the material to be studied. A stress testing instrument with multi-point bending modes capable of applying controlled loads to the ceramic piece under study is a reliable approach to determining the relationship between the Raman shift and stress [23,24,25]. It is also possible to perform these measurements thanks to a high-pressure diamond anvil cell [26,27]. This equipment can create a high hydrostatic pressure of several hundred gigapascals.

To date, and as far as the authors have found in the literature, no study has been carried out under the conditions of a Hertz mechanical contact to determine the relationship between the Raman band shift of the material and the contact pressure. The advantage of this situation is that it is analytically well described [28]. Additionally, this measurement can be a preliminary step in tribological studies. More precisely, in the contact between a sphere with the radius *R* and a plane, the contact area is circular with the radius *a*. The pressure distribution in the contact is depicted according to Relation (1).

(1) $$\begin{array}{c} p\left(r\right)=p_{0}\sqrt{1-{\left(\frac{r}{a}\right)}^{2}} \end{array}$$

**Figure 1:**
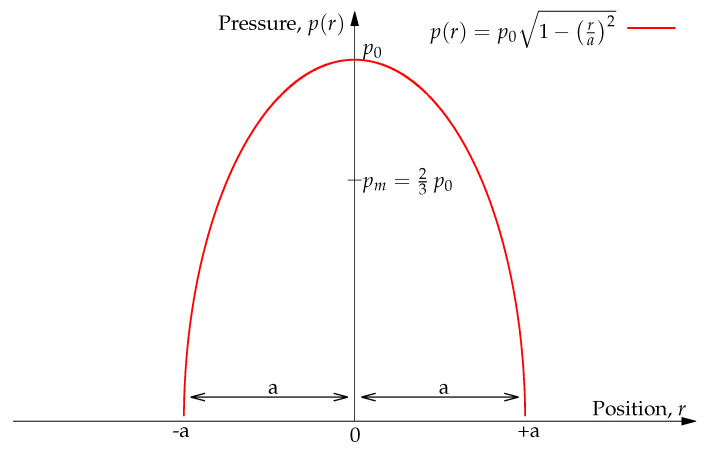
Pressure distribution in Hertzian contact. The maximum pressure is denoted by $p_{0}$, and the mean pressure is denoted by $p_{m}$. The distribution is centred at zero and spans over the contact’s radius, *a*. Beyond the contact radius (r>a), the distribution is zero.

In this equation, *r* specifies the position considered. The pressure is maximum at r=0 and noted as $p_{0}$. The average pressure $p_{m}$ corresponds to 2/3 of the maximum pressure $p_{m}$ (Figure 1).

Thus, the total load $F_{N}$ is such that:

(2) $$\begin{array}{c} F_{N}=\int_{0}^{a}p\left(r\right)\;2\;\pi \;rdr=\frac{2}{3\;}p_{0}\;\pi \;a^{2} \end{array}$$

It is possible to express the contact radius *a* as a function of the radius of the sphere *R* and the applied force $F_{N}$, as follows:

(3) $$\begin{array}{c} a={\left(\frac{3\;F_{N}\;R}{4\;E^{*}}\right)}^{\frac{1}{3}} \end{array}$$

The equivalent modulus $E^{*}$ (Equation (4)) is expressed as a function of Young’s moduli $E_{1}$ and $E_{2}$ and the Poisson coefficients $\nu_{1}$ and $\nu_{2}$ of the two considered materials:

(4) $$\begin{array}{c} \frac{1}{E^{*}}=\frac{1-\nu_{1}^{2}}{E_{1}}+\frac{1-\nu_{2}^{2}}{E_{2}} \end{array}$$

However, this approach requires that the following assumptions, made by Hertz in his theory, be respected:

- The materials are homogeneous and elastic. They are considered semi-infinite;
- Their surfaces are continuous;
- Their geometries do not conform;
- The dimensions of the materials are large compared to the dimensions of the contact;
- There is no friction and no adhesive force.

In this paper, we propose to determine the relationship between the Raman band shift and the contact pressure thanks to a Hertzian contact, realised by the static contact between a ball and a plane.

The ceramics allow us to approach the homogeneity and elasticity conditions of the model proposed by Hertz. We selected two ceramics to build our contact: a polycrystalline alumina ball and a soda–lime glass plane. Alumina has well-identified Raman modes. Early work in Raman spectroscopy focused on corundum, sapphire and ruby, which are single-crystal forms of alumina [29,30,31,32].

**Figure 2:**
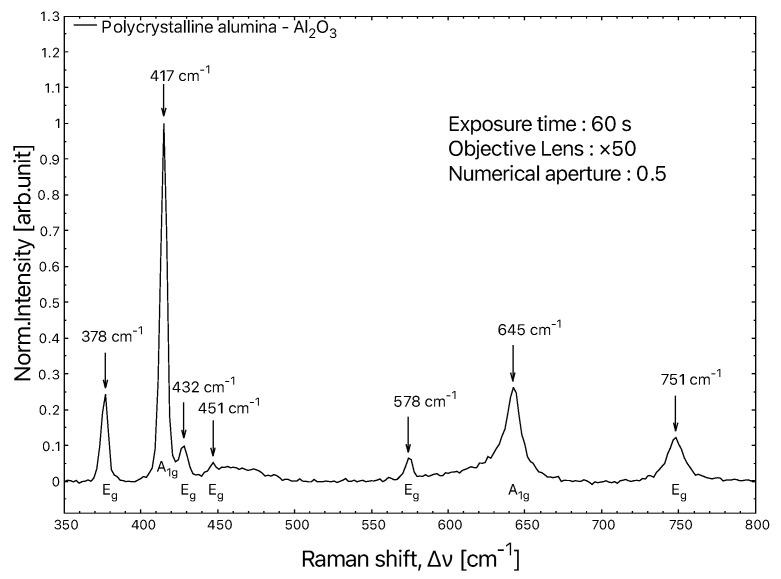
Typical Raman spectrum of polycrystalline alumina. The two modes at 417 cm${}^{-1}$ and 645 cm${}^{-1}$ are of type $A_{1g}$. The five modes at 378 cm${}^{-1}$, 430 cm${}^{-1}$, 451 cm${}^{-1}$, 576 cm${}^{-1}$ and 750 cm${}^{-1}$ have $E_{g}$ symmetry. The spectrum is normalised to the strongest peak at 417 cm${}^{-1}$.

They are grouped in two irreducible representations: $A_{1g}$ and $E_{g}$. The $A_{1g}$ symmetry contains two bands at around 417 cm${}^{-1}$ and 645 cm${}^{-1}$, while the $E_{g}$ symmetry contains five bands around 378 cm${}^{-1}$, 430 cm${}^{-1}$, 451 cm${}^{-1}$, 576 cm${}^{-1}$ and 750 cm${}^{-1}$. The Raman peaks are predominantly narrow and intense, which makes them easy to record and process (Figure 2).

The literature contains studies of band displacement as a function of pressure. The authors were initially interested in single-crystal alumina under either tensile or compressive stress. Firstly, there is the seminal work of Watson et al. with a high-pressure cell ranging from 0.1 to 1 GPa of compression [32] and of Jia and Yen with sapphire fibres under tensile stress up to 0.65 GPa [33]. Gallas et al. studied the modes at 378 and 417 cm${}^{-1}$ using a diamond anvil with isostatic pressures up to 3.2 GPa [27]. Kimashi et al. studied the displacement of all modes with a four-point bending test [23], except for the 451 cm${}^{-1}$ mode. More recently, in 2021, Akatsu et al., with a four-point bending test, refined the value of the displacement coefficient under tensile stress [25]. Pezzoti and Zhu treated both theoretically and experimentally the case of polycrystalline alumina. From the polarised Raman measurements, they extracted all the components of the stress tensor [24].

Glass is a solid, usually transparent material. It is prepared from the fusion of silica sand. It is amorphous silica whose melting temperature is high, around 1723 °C [34], and its structure comprises disordered SiO${}_{4}$ tetrahedrons [35]. In the silica glass lattice, there are only siloxane bonds, O-Si-O. All oxygen atoms are bonded to two silicon atoms. The oxygen atoms are at the vertices of a tetrahedral site whose centre is occupied by a silicon atom. The SiO${}_{4}$ tetrahedra are denoted Q${}^{i}$. The exponent *i*, which varies from 0 to 4, represents the number of oxygen atoms that bridge with another tetrahedron. There can, therefore, be up to four bridging oxygen atoms in a tetrahedron. Seven bridges denoted as Q${}^{i}$-O-Q${}^{j}$ are identified between the SiO${}_{4}$ tetrahedra in silica glass with i,j=1−4 and |i−j|<2 [36,37].

The glass’s properties can be controlled to reduce the melting temperature. For example, alkali or alkaline earth elements can be added during the glass preparation. It is then possible to manufacture binary glasses [34,38,39], ternary glasses [40,41] or more complex glasses [42]. The structure of these glasses then contains non-bridging oxygen atoms, which do not connect two SiO${}_{4}$ tetrahedra. In this case, the non-binding oxygen is associated with an alkaline or alkaline–earth atom. The bond is then denoted as X-O-Q${}^{n}$ in which n=0−3, and *X* is the alkaline or alkaline–earth atom [36].

The glass used in this study is a soda–lime glass commonly used for microscopy slides. These glasses have a complex composition. We could have chosen a more suitable transparent material, but this glass has the advantage of being readily available. It can be easily replaced if it breaks during a plane compression test. It consists of the ternary system CaO-Na${}_{2}$O-SiO${}_{2}$.

Raman spectroscopy is regularly used to study glasses for fundamental or applied research, earth sciences or cultural legacies [43,44]. The spectrum of silica glass and those of binary, ternary and quaternary silicate glasses are decomposed into four parts [34,45,46]:

- The boson peak is between 10 and 250 cm${}^{-1}$;
- The low-frequency region is between 250 and 700 cm${}^{-1}$;
- The intermediate region is between 700 and 850 cm${}^{-1}$;
- The high-frequency region is between 850 and 1300 cm${}^{-1}$.

In the case of soda–lime glasses, the spectra reveal bands around 450 and 560 cm${}^{-1}$ associated with the symmetric stretching modes of the Q${}^{4}$ and Q${}^{3}$ species. According to the authors, the band around 600 cm${}^{-1}$ is assigned either to the symmetric tetrahedron stretching mode of Q${}^{2}$ or to the Si three-membered ring breathing mode also found in pure silica glass and annotated D${}_{2}$. Si oscillations in the octahedral site cause the mode to appear at 800 cm${}^{-1}$. Finally, the bands around 950 and 1100 cm${}^{-1}$ are related to the stretching modes of the Si-O bonds in the Q${}^{2}$ and Q${}^{3}$ tetrahedra, respectively [47,48,49,50].

Under isostatic pressure, soda–lime glasses undergo elastic deformation up to a limit of 7 GPa. Above 16.2 GPa, permanent deformation is observed. The bands are modified by compression, and the centroids of the peaks move towards high frequencies [48,49].

**Figure 3:**
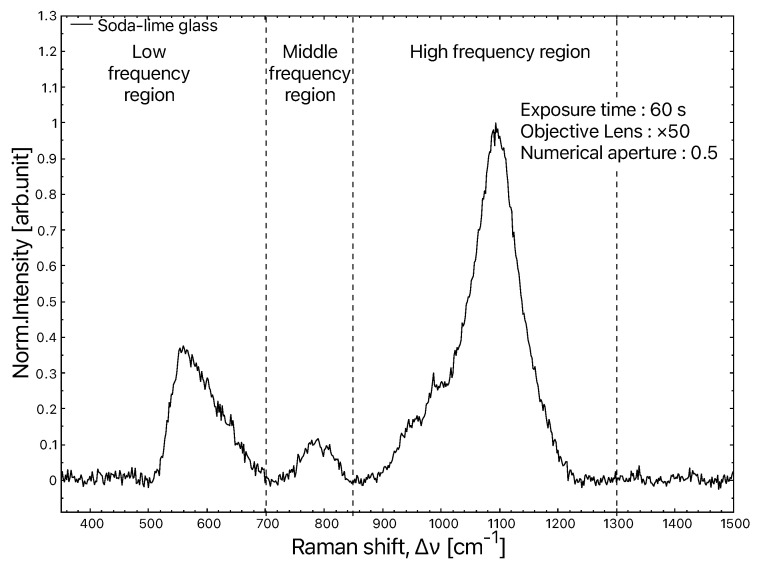
Typical Raman spectrum of soda–lime glass.

In the present study, we propose to verify the pressure-dependent band-shift relationships under the conditions of a Hertz contact between a polycrystalline alumina ball and a transparent soda–lime glass plate (Figure 3). We also plan to measure the pressure profile in the contact interface locally.

Section 2 presents the experimental approach and the approach used to trace the relationship between the Raman band shift and the pressure. Section 3 presents the results obtained for different loading conditions, i.e., the pressure values measured optically, rather than those obtained by Raman spectroscopy. Finally, we propose a discussion (Section 4) highlighting our results against those previously acquired with diamond anvils or four-point bending tests.

## 2. Materials and Methods

Alumina is polycrystalline with a purity of 99.80%. It is white in colour. The ball has a radius of 5 mm and a surface roughness of 0.5 µm. Polycrystalline alumina’s Young’s modulus is 380 GPa, and its Poisson’s ratio is 0.3. The soda–lime glass slide is transparent. Its dimensions are standard (ISO 8037/1) with a width of 26 mm and a length of 76 mm. Its thickness equals 1 mm, and its surface roughness is 0.5 µm. Soda–lime glass’s Young’s modulus is 70 GPa, and its Poisson’s ratio is 0.16.

**Figure 4:**
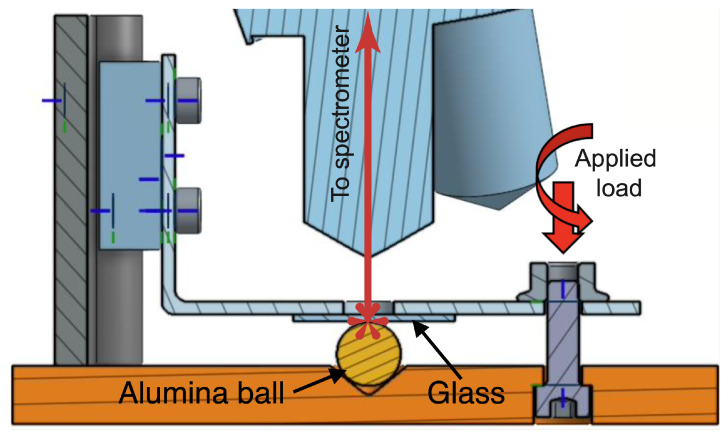
The instrument for compressing the alumina ball and the glass plate.

The experimental campaign was conducted by making a Hertz contact between the alumina ball and the glass plate. The ball and the plate were assembled in an instrument that ensures stable compression and has an opening to observe the interfacial contact through the glass slide (Figure 4). Thirteen loading conditions are presented in this article.

The spectra were recorded using a Renishaw InVia Qontor Raman microscope equipped with a laser wavelength of 532 nm and a nominal power of 50 mW. The microscope has an objective of ×50, a numerical aperture of 0.5 and a long working distance of 8.2 mm. Under these conditions, the lateral resolution (size of the laser spot) is 1.3 µm, its axial resolution Δz is 2.3 µm, and the power measured at the microscope’s output is 5.8 ± 0.5 mW. The spectra were recorded for 60 s and accumulated three times. Using a motorised table, the Raman spectra profiles were recorded on a segment that passes through a contact diameter for each loading condition. The segment has a length of 500 µm. The accuracy of the displacement is 0.05 µm. The spectra were recorded every 2 µm.

The 251 spectra were processed after registration:

- The spectra were truncated from 300 to 1300 cm${}^{-1}$;
- The cosmic peaks were eliminated;
- The signal-to-noise ratio was reduced by filtering;
- The spectra were normalised using the strongest alumina peak at 417 cm${}^{-1}$.

The peaks associated with alumina and glass were then fitted using information from the literature and the Renishaw Wire 5 software. Their positions and widths at half height are detailed in Table 1 and Table 2. These fits of the vibration modes allow the displacement of the alumina and glass Δν bands to be extracted as a function of *r*, the position along the spectral profile. The baseline was treated during the fitting of each spectrum.

The pressure profile equation p(r) (Equation (1)) from the Hertz model is a function of the maximum pressure $p_{0}$, the contact radius *a*, and the position on the contact profile *r*. This function is even, defined between −a and +a, and p(r) varies between 0 and $p_{0}$. From Equation (1), we deduce empirically Equation (5) of the spectral profile, which follows the pressure variation and can be described by the Hertz model:

(5) $$\begin{array}{c} \Delta \nu \left(r\right)=\Delta \nu_{max}\sqrt{1-{\left(\frac{r}{a}\right)}^{2}} \end{array}$$

We inject 2 terms: $\Delta \nu_{0}$ and $r_{0}$. The term $\Delta \nu_{0}$ adds a continuous component to the function since the offset Δν(r) is not zero beyond the contact radius. The term $r_{0}$ allows for the centring of the contact, which is not necessarily perfect when the sample is placed under the microscope. Then, Equation (5) becomes:

(6) $$\begin{array}{c} \Delta \nu \left(r\right)=\Delta \nu_{max}\sqrt{1-{\left(\frac{r-r_{0}}{a}\right)}^{2}}+\Delta \nu_{0} \end{array}$$

So, the Δν(r) profile is fitted to a curve similar to the Hertz profile (Equation (6)) thanks to Gnuplot software [51]. Thanks to this adjustment, the contact radius *a* and the amplitude $\nu_{max}$ are deduced. Equations (2) and (3) allow us to calculate the maximum pressure $p_{0}$ from *a* with Young’s moduli and Poisson’s ratios of the glass and polycrystalline alumina. The parameters $r_{0}$ and $\Delta \nu_{0}$ participate in the curve fitting by shifting the profile along the x-axis and y-axis, respectively.

(7) $$\begin{array}{c} p_{0}=\frac{2\;a\;E^{*}}{R\;\pi } \end{array}$$

Calculating the pressure for all measured points from Equation (2) is possible. Finally, the relationship between the Raman mode shift and the Hertz pressure Δν(p) of interest is plotted.

## 3. Results

### 3.1. Optical Measurements

**Figure 5:**
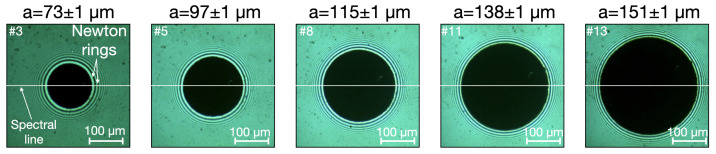
Images of the contact: Images from the microscope are displayed with a scale of 100 µm in presentation software. A circle with a thickness of 0.75 pt to measure the diameter of the contact disc is drawn without filling. Given our scale of 100 µm or 85.32 pt, the thickness of the circle is 0.88 µm. Since the diameter is measured from edge to edge of the circle, the uncertainty in the diameter measurement is twice the thickness of the circle line, i.e., 1.76 µm. Consequently, the uncertainty in determining the radius is divided by two, i.e., 0.88 µm. Finally, this value is rounded to 1 µm to be used as the uncertainty of the radius.

Images of the contact and the measurement of the contact diameter by optical microscope are presented in this section (Figure 5). The contact appears as a dark disc. Newton’s rings surround it because of the air gap between the ball and the plane beyond the contact, which causes interference fringes.

The images allow the contact radius to be measured with an uncertainty of about 1 µm. The white line indicates the position of the segment along which the spectra are measured. The maximum pressure $p_{0}$ within the thirteen loading conditions is then calculated with Equation (7). The results are given in Table 3. The contact radius varies from 47 µm in the first test to 155 µm in the last. The maximum pressure evolves from 0.37 GPa to 1.17 GPa, respectively.

### 3.2. Raman Measurements

**Figure 6:**
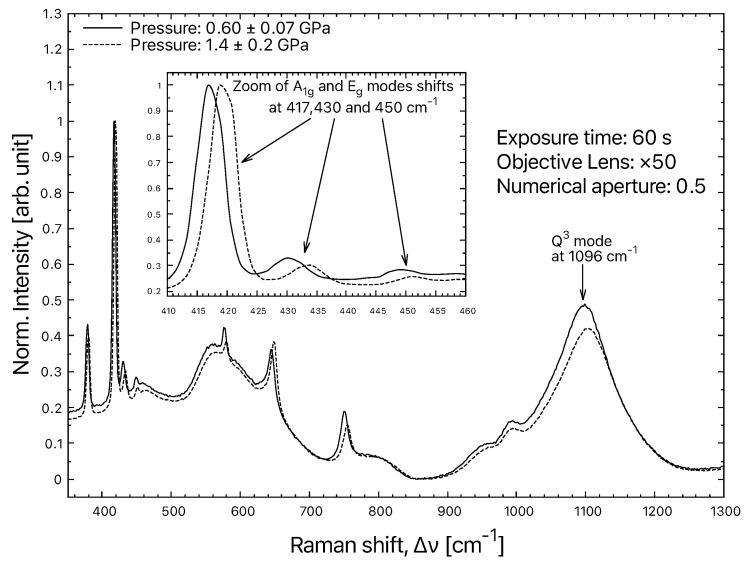
The shift of the Raman spectrum was recorded at the alumina–glass interface for two pressure conditions: at 0.6 GPa (solid line) and 1.4 GPa (dashed line). The enlargement shows the region between 410 and 440 cm${}^{-1}$.

The results in this section detail the data acquired for the polycrystalline alumina mode and the soda–lime glass. After processing the spectra, the band shifts for the two extreme conditions show the effect of compression on the band positions of both the alumina and glass bands in Figure 6.

**Figure 7:**
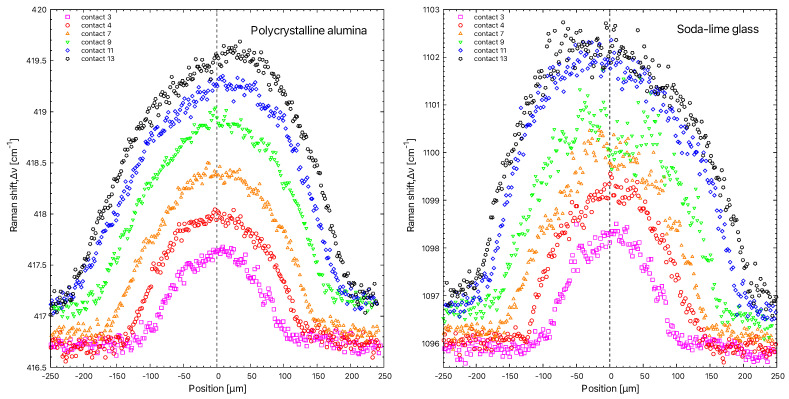
Raman band shift as a function of contact position for polycrystalline alumina (**left**) and soda–lime glass (**right**).

The spectrum measured at a maximum pressure of 0.6 GPa is shifted compared to the spectrum measured at a pressure of 1.4 GPa. The wave numbers of the vibrational modes move significantly to the right. To quantitatively measure the dependence of the Raman mode shift on the pressure, we look at the most intense modes of each material: the one at 417 cm${}^{-1}$ and the one from the soda–lime glass at 1096 cm${}^{-1}$. The Raman wave number shifts Δν are displayed as a function of the position in the contact (Figure 7). They are calculated from the curves’ analyses and the 417 cm${}^{-1}$ mode and 1096 cm${}^{-1}$ mode deconvolutions.

Several spectral profiles were represented for different contact situations. Note that the profile has a bell shape comparable to that of the Hertz pressure profile. As expected from the theory, the base of the pressure profile increases with increasing pressure. The top of the curve also increases with increasing stress. It is noticeable that the profile is sometimes irregular. Furthermore, at higher pressures, the spectral profile tends to become asymmetric. For the alumina, the shift occurs to the right, while for the soda–lime glass it occurs to the left.

### 3.3. Contact Radii and Pressures

The fitting of the spectral profiles with Equation (6) allows us to deduce the contact radii. The values of $\Delta \nu_{max}$, the radius deduced by the Raman spectroscopy noted as ${}^{R}a$, and those of the maximum pressure calculated from the spectral profile, noted as ${}^{R}p_{0}$, are listed in Table 4.

**Figure 8:**
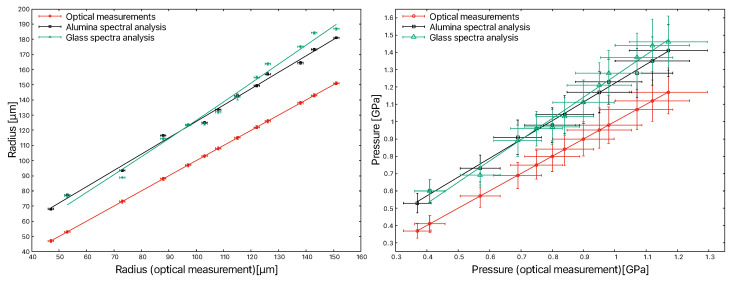
Comparison of the contact’s radius measurements (**left**) and the maximum pressure measurements (**right**) depending on the method used.

The spectral profile of the glass in the first contact was not exploited, as the signal-to-noise ratio was too bad. In the polycrystalline alumina, the radii range from 68 µm to 181 µm, while in the soda–lime glass, the radii increase from 77 µm to 187 µm. The maximum pressures measured in the different contacts range from 0.53 GPa to 1.46 GPa. The relative difference between the measurements made with the alumina and glass is about 3.2% on average. The differences between the measurements made with the optical microscope and the Raman spectra are considerable. These can also be seen in Figure 8. These relative differences are of the order of 26% on average.

### 3.4. Relationship between Contact Pressure and Raman Shift

From the maximum pressure values, ${}^{R}p_{0}$, and the previously obtained contact radius ${}^{R}a$, it is now possible to know the pressure for each point in all the contacts considered.

**Figure 9:**
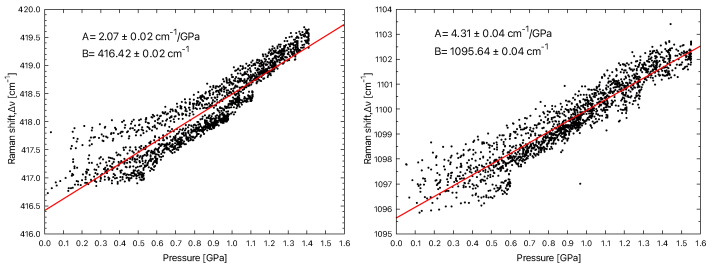
Relationship between contact pressure and Raman shift of the $E_{g}$ mode of alumina at 417 cm${}^{-1}$ (**left**) and the Q${}^{3}$ mode of soda–lime glass at 1095 cm${}^{-1}$ (**right**).

For this, we use Equation (1). It is, therefore, possible to represent the contact pressure as a function of the $E_{g}$ mode shift of the alumina (Figure 9 on the left) and the soda–lime glass (Figure 9 on the right).

A linear fit is used to describe the trend and extract the coefficient. The uncertainty corresponds to asymptotic standard errors. We thus find the value of 2.07 ± 0.02 cm${}^{-1}$/GPa, which is comparable to the values deduced by Gallas et al. Indeed, according to these authors, the wave number shifts with the pressure were calculated in hydrostatic and non-hydrostatic pressure domains. They were, respectively, between 1.97 ± 0.08 cm${}^{-1}$/Gpa and 2.20 ± 0.07 cm${}^{-1}$/GPa. However, in contrast to these authors, the linear regression results in a higher origin order of about 416 cm${}^{-1}$, instead of 418 cm${}^{-1}$. In the case of soda–lime glass, we calculate a coefficient of the order of 4.31 ± 0.04 cm${}^{-1}$/GPa with an intercept of 1095.64 cm${}^{-1}$.

## 4. Discussion

We have performed Raman spectral profiles in contact between an alumina sphere and a glass plane. The spectral profiles allowed us to deduce the contact size, the maximum pressure and, after all, the pressure dependence of the wave number $\frac{d\nu }{dp}$ of the more intense Raman modes for the two materials studied.

In the case of polycrystalline alumina, the value of the pressure dependence of the 417 cm${}^{-1}$ that we deduced is in good agreement with those obtained by other methods and already published in the literature [27,32].

In the case of soda–lime glass, we worked in a 0.37 to 1.46 Gpa pressure range that had yet to be explored [48,50]. As a result, we were able to provide an accurate value for the Q${}^{3}$ mode’s pressure dependence on this pressure range.

Our method is relatively simple and inexpensive compared to a high-pressure diamond anvil cell. It allows many measurements to be made in a single test over a finely graded pressure range. Moreover, it is easily reproducible with reasonable accuracy.

The mode displacements as a function of the pressure $\frac{d\nu }{dp}$ are deduced independently for the two materials tested. However, as in the case of diamond anvils, one of the two materials can be used as a standard and can act as a control for the material with the unknown $\frac{d\nu }{dp}$ relationship. This is the role of the ruby splinters that are sometimes added in some diamond anvils to check the pressure intensity [26].

We found that the contact diameters determined by Raman spectroscopy were systematically larger than those measured by optical microscopy. This suggests that optical measurements differ from spectral measurements, probably due to refraction phenomena and deformation of the glass plate under stress. Illumination and interference rings can also limit the quality of the measures. It would be interesting to investigate and compare several methods for measuring other contact features, such as the contact radius, to better assess and understand the differences between these methods.

In the case that the pressure dependence of the wave number is known for both materials, it may become interesting to use our backward method to deduce Young’s moduli and Poisson’s ratios. This is possible since Equations (3) and (4) depend on the E and ν of the two materials. This also reveals one of the drawbacks of our approach: the accuracy of the results is strongly associated with the knowledge of Young’s moduli and Poisson’s ratios.

Furthermore, the measurement conditions must remain within the framework of the Hertz model, so the method is unsuitable for materials that have reached their plasticity limit. This can happen for metals that are rarely active in Raman spectroscopy, polymers and their composites. In the case of polymers, it is necessary to consider their plasticity and adhesion. In the future, we plan to implement mechanical contact models that take plastification into account to study the compression of polymers beyond Hertz conditions [28]. We will also look at adhesion with models such as JRK and DMT [52,53,54].

In the case of ceramics, although we are within the desired elasticity conditions of the Hertz model, we may reach the fracture limit of the parts. This limit considerably reduces the range of pressure under which the tests are performed. In our tests, the fracture limit of the materials leads to the limit of the studied pressure range, which is different in the diamond anvils, therefore allowing the experimental study and even the plastification [48,50].

Still, following the Hertz model, the surface must be perfectly continuous and smooth, and no friction may occur. The presence of friction or asperities in real contact can produce a shift in the profile [28]. In our tests, for the highest pressures, the spectral profile deviates from the Hertz profile. A misalignment of our compression instrument may also explain this asymmetry.

On the other hand, we have noted the presence of two populations of points during the exploitation of the $\frac{d\nu }{dp}$ relations. This is well observed in the case of polycrystalline alumina in Figure 9 (left). However, these two populations of points do not affect the coefficient value obtained from the pressure gradient and the band offset gradient. These two populations are perhaps due to the mechanical backlash of the compression system, which modifies the stress field around the contact. We have studied the case in which we increase the pressure, but we have not evaluated the influence of the discharge or the charge/discharge cycling to verify this hypothesis. This could be the subject of future work.

We plan to exploit this method to study dynamic contacts in fretting, i.e., alternating translations shorter than the contact diameter or reciprocating sliding. The dissipative phenomenon, typical of tribological experiments without lubrication, is then able to be explored using operando Raman spectroscopy. Moreover, this method may be able to provide essential data on pressure and temperature gradients and help elucidate the mechanisms that occur in these dynamic interfaces. 

## Figures and Tables

**Table 1:** Positions, full width at half maximum and intensity of the polycrystalline alumina bands used to fit the spectra.

Band	FWHM	Intensity	Symmetry
(cm${}^{-1}$)	(cm${}^{-1}$)	(norm.)	or Assignment
378	5.2	0.28	E${}_{g}$
417	5.3	1	A${}_{1g}$
430	7.1	0.11	E${}_{g}$
450	4.7	0.03	E${}_{g}$
577	12.0	0.10	E${}_{g}$
645	14.2	0.27	A${}_{1g}$
751	13.8	0.13	E${}_{g}$

**Table 2:** Position, full width at half maximum and intensity of the soda–lime glass bands used to fit the spectra. Intensities are normalised to the most intense alumina peak at 417 cm${}^{-1}$. Sym. stretch.: symmetrical stretching. Si-O stretch: stretching of Si-O bonds.

Band	FWHM	Intensity	Symmetry
(cm${}^{-1}$)	(cm${}^{-1}$)	(norm.)	or Assignment
458	27.5	0.05	Q${}^{4}$ - Sym. strech.
554	34.5	0.17	Q${}^{3}$ - Sym. strech
600	82.2	0.20	Q${}^{2}$ - Sym. strech
789	48.0	0.06	Si oscillations
939	35.5	0.06	Q${}^{2}$ - Si-O strech.
991	68.1	0.15	Q${}^{2}$ - Si-O strech.
1096	97.9	0.70	Q${}^{3}$ - Si-O strech.

**Table 3:** Radius ${}^{O}r$ and maximum contact pressure ${}^{O}p_{0}$ measured from the optical micrographs in the different contacts. The uncertainty of the radius is 1 µm.

Contact	Radius ${}^{O}r$	Max. Pressure ${}^{O}p_{0}$
Number	in µm	in GPa
1	47	0.37 ± 0.05
2	53	0.41 ± 0.05
3	73	0.57 ± 0.07
4	88	0.69 ± 0.08
5	97	0.75 ± 0.09
6	103	0.80 ± 0.09
7	108	0.84 ± 0.10
8	115	0.90 ± 0.10
9	122	0.95 ± 0.11
10	126	0.98 ± 0.11
11	138	1.07 ± 0.12
12	143	1.12 ± 0.12
13	151	1.17 ± 0.13

**Table 4:** Maximum Raman shift $\Delta \nu_{max}$, radii ${}^{R}a$ and maximum contact pressures ${}^{R}p_{0}$ deduced from spectral profiles.

	Polycrystalline Alumina			Soda–Lime Glass		
**Contact**	$\Delta \nu_{\max}$	${}^{R}a$	${}^{R}p_{0}$	$\Delta \nu_{\max}$	${}^{R}a$	${}^{R}p_{0}$
**Number**	**in cm** ${}^{-1}$	**in µm**	**in GPa**	**in cm** ${}^{-1}$	**in µm**	**in GPa**
1	0.31 ± 0.02	68.0 ± 0.5	0.53 ± 0.06	-	-	-
2	0.51 ± 0.02	77.3 ± 0.6	0.60 ± 0.07	1.20 ± 0.02	76.8 ± 0.9	0.60 ± 0.06
3	0.82 ± 0.02	93.5 ± 0.5	0.73 ± 0.08	2.23 ± 0.03	88.8 ± 0.5	0.69 ± 0.07
4	1.18 ± 0.02	116.6 ± 0.7	0.91 ± 0.10	3.03 ± 0.04	114.4 ± 0.7	0.89 ± 0.09
5	1.26 ± 0.02	123.4 ± 0.6	0.96 ± 0.10	3.25 ± 0.02	123.6 ± 0.8	0.96 ± 0.10
6	1.27 ± 0.02	125.0 ± 0.9	0.98 ± 0.10	3.17 ± 0.05	124.3 ± 0.9	0.97 ± 0.10
7	1.45 ± 0.02	133.5 ± 0.7	1.04 ± 0.11	3.54 ± 0.04	132.0 ± 0.7	1.03 ± 0.11
8	1.51 ± 0.02	142.6 ± 0.9	1.11 ± 0.12	3.46 ± 0.12	142.0 ± 2.0	1.11 ± 0.13
9	1.64 ± 0.02	149.4 ± 0.7	1.17 ± 0.12	3.85 ± 0.06	155.0 ± 0.8	1.21 ± 0.13
10	1.74 ± 0.03	157.1 ± 0.7	1.23 ± 0.13	4.27 ± 0.05	163.8 ± 0.7	1.28 ± 0.13
11	1.89 ± 0.03	164.4 ± 0.9	1.28 ± 0.14	4.91 ± 0.05	175.2 ± 0.7	1.37 ± 0.14
12	2.02 ± 0.03	173.3 ± 0.8	1.35 ± 0.14	4.97 ± 0.06	184.2 ± 0.8	1.44 ± 0.15
13	2.17 ± 0.03	181.0 ± 0.7	1.41 ± 0.15	4.91 ± 0.06	186.9 ± 0.8	1.46 ± 0.15

## Data Availability

The data presented in this study are available on request from the corresponding author.
